# Bottom-Up Self-Assembly Based on DNA Nanotechnology

**DOI:** 10.3390/nano10102047

**Published:** 2020-10-16

**Authors:** Xuehui Yan, Shujing Huang, Yong Wang, Yuanyuan Tang, Ye Tian

**Affiliations:** 1 College of Engineering and Applied Sciences, State Key Laboratory of Analytical Chemistry for Life Science, Nanjing University, Nanjing 210023, China; MG1934036@smail.nju.edu.cn (X.Y.); DZ1934015@smail.nju.edu.cn (S.H.); DG20340046@smail.nju.edu.cn (Y.W.); MF20340057@smail.nju.edu.cn (Y.T.); 2 Shenzhen Research Institute of Nanjing University, Shenzhen 518000, China; 3 Chemistry and Biomedicine Innovation Center, Nanjing University, Nanjing 210023, China

**Keywords:** self-assembly, bottom-up, DNA tile, DNA brick, DNA origami, nanoparticles

## Abstract

Manipulating materials at the atomic scale is one of the goals of the development of chemistry and materials science, as it provides the possibility to customize material properties; however, it still remains a huge challenge. Using DNA self-assembly, materials can be controlled at the nano scale to achieve atomic- or nano-scaled fabrication. The programmability and addressability of DNA molecules can be applied to realize the self-assembly of materials from the bottom-up, which is called DNA nanotechnology. DNA nanotechnology does not focus on the biological functions of DNA molecules, but combines them into motifs, and then assembles these motifs to form ordered two-dimensional (2D) or three-dimensional (3D) lattices. These lattices can serve as general templates to regulate the assembly of guest materials. In this review, we introduce three typical DNA self-assembly strategies in this field and highlight the significant progress of each. We also review the application of DNA self-assembly and propose perspectives in this field.

## 1. Introduction

The arrangement of individual atoms or molecules determines the physical and chemical properties of a material. If these properties can be manipulated precisely at the atomic level, a variety of precious materials with novel physical or chemical properties can be synthesized. The properties of materials can be tailored specifically with a given application; however, thus far, this remains a huge challenge in the fields of chemistry and material science. DNA nanotechnology offers the possibility of customizing the properties of materials. In the 1980s, Professor Seeman first proposed using DNA strands to self-assemble from the bottom-up into three-dimensional (3D) crystals [1]. DNA 3D crystals can serve as a universal platform for organizing biological and inorganic nanocomponents into 3D arrays. This idea is generally considered the origin of DNA nanotechnology. Structural DNA nanotechnology focuses on the programmability and specificity of DNA molecules, and aims to assemble DNA structures into crystals or to guide the crystallization of other components [2,3,4]. Some results show that this technology can achieve precise control of materials in nano-scale [5].

In order to achieve the refined control and synthesis of more complex structures, several effective assembly methodologies have been proposed, including DNA tile [6], DNA brick [7], and DNA origami [8]. A tile is a complex of DNA branching motifs self-assembled from short single-stranded DNA molecules. By carrying single-stranded DNA as a sticky end to link with other specific units, tiles can further self-assemble into two-dimensional (2D) arrays [6,9], 3D lattices [10], or polyhedral framework structures [11]. DNA bricks can be regarded as the expansion of tiles from 2D to 3D, but this method can construct 3D structures of any shapes with higher designability [7,12]. In general, tiles and bricks focus on using small building blocks to arrange large periodic structures, but the geometric features of a single building block are relatively simple. The complex features of a single building block can be achieved by DNA origami. In 2006, Rothemund proposed a new method of DNA self-assembly called DNA origami [8]. A scaffold chain containing thousands of bases is folded into arbitrary shape, with the help of hundreds of short single-stranded oligonucleotides which are called “staples.” Since each staple strand is different, some can be designed as sticky ends that are located on the periphery of the final assembly. The structural features of origami endow the sticky ends addressability and directionality, allowing origami to accurately capture and organize other nano-objects. Using DNA origami, a large number of nanostructures with prescribed shapes and sizes have been constructed. In addition, monodisperse structures can be further assembled into large periodic lattices [2,13,14,15]. For example, Tian et al. assembled polyhedral origami structures into multiple superlattice frameworks and co-crystallized them with nanoparticles of different compositions [2,14]. Although these self-assembly methods are slightly different in assembly methods and purposes, the goals of uniform development are to improve assembly controllability and to build more complex structures.

Since its development, each DNA-based self-assembly method has experienced multiple technological breakthroughs. For this review, we selected three more mature DNA self-assembly methods, namely DNA tile, DNA brick, and DNA origami. Herein, we analyze the technological breakthroughs and major achievements of each self-assembly method in the development process from both theoretical and experimental aspects. The application of each self-assembly method in manipulating object materials is also included. Finally, we discuss the challenges and prospects that still exist in the field of structural DNA nanotechnology.

## 2. Self-Assembly Based on DNA Tile

**Figure 1:**
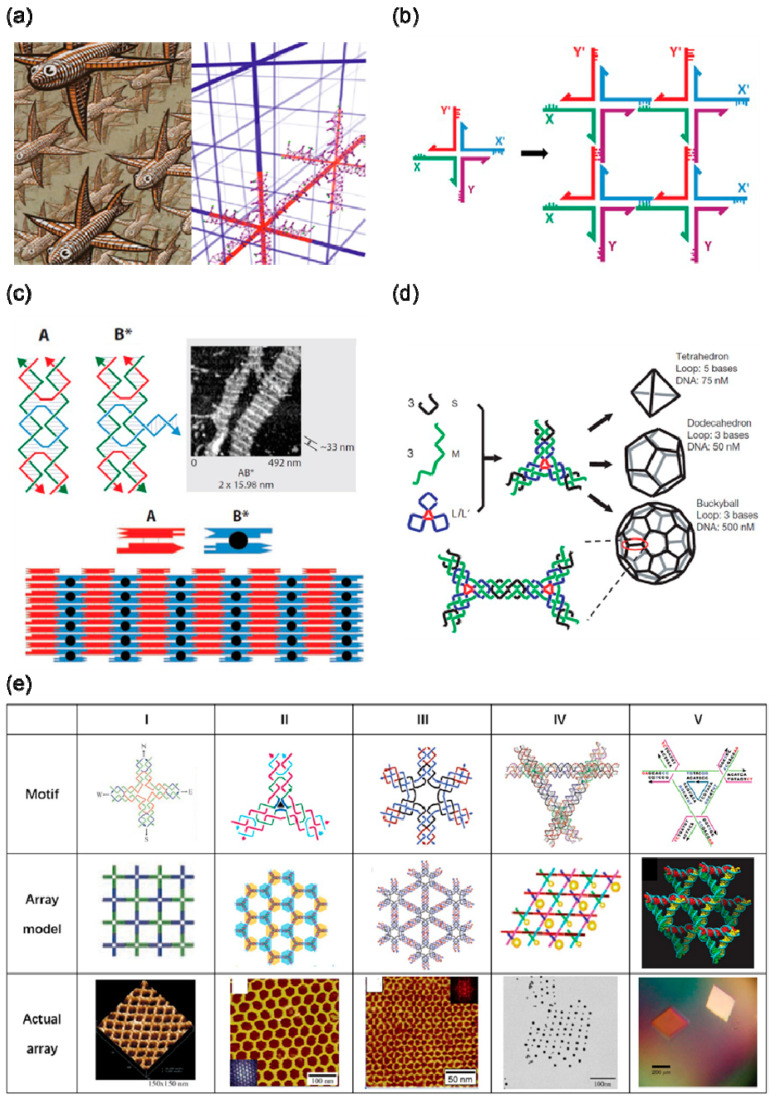
Self-assembly based on DNA tiles (**a**) Escher’s woodcut depth (**left**) and the prototype of DNA nanotechnology inspired by it (**right**). Reproduced from [21]. (**b**) A schematic diagram of a two-dimensional (2D) DNA lattice assembled by four-arm holiday junctions connected by sticky ends. Reproduced with permission [22]. Copyright Materials Research Society, 2017. (**c**) A 2D lattice assembled by DNA double-crossover molecule (DX) and DX + J tiles. Reproduced with permission of [22]. Copyright Materials Research Society, 2017. (**d**) The structure of the DNA polyhedron assembled by three-pointed star DNA motifs. Reproduced with permission of [11]. Copyright Springer Nature, 2008. (**e**) DX-based complex DNA tile motif library and its assembly results. (I) 4 × 4 tile. Reproduced with permission of [9]. Copyright American Association for the Advancement of Science, 2003; (II) three-pointed star. Reproduced with permission of [23]. Copyright American Chemical Society, 2005; (III) six-pointed star. Reproduced with permission of [24]. Copyright American Chemical Society, 2006; cop; (IV) DX-based tensegrity triangle. Reproduced with permission of [25]. Copyright American Chemical Society, 2006; (V) a tensegrity triangle with complete triple symmetry. Reproduced from [10].

In 1982, Seeman proposed using DNA to construct a 3D periodic network, which is considered to be the origin of “DNA nanotechnology” (Figure 1a) [1]. In the early years, DNA branched junctions were selected as basic building blocks for constructing 3D periodic networks, as shown in Figure 1b. In 1983, Seeman et al. successfully synthesized the four-arm junction structure of DNA [16]. DNA building blocks are required to have a certain rigidity for assembling into a larger structure, but DNA branched junctions obviously fail to meet this feature. By introducing a crossover between two DNA double helices, Seeman et al. designed the “DNA double-crossover molecule” (DX) with sufficient rigidity to form a larger structure [17]. In the mathematical theory of tiling, rectangular tiles with programmable interactions, known as Wang tiles, can be tiled into 2D spaces. DX molecules can be arranged in a 2D space imitating a Wang tile, and single-stranded DNA sequences can be extended on both sides of the DX molecule as sticky ends to connect other DX molecules. Reasonably designed sticky ends can assemble DX molecules and DX + J molecules into a 2D array (Figure 1c) [6,18]. The structure of a single DNA tile is relatively simple, consisting of several stoichiometric single oligonucleotide strands. Simple small units are repeatedly arranged to form a large 2D array through the connection of sticky ends. However, this method has relatively low control over the size and shape of the product. Li et al. proposed to restrict the size and shape of DNA tile growth with a prescribed DNA origami frame [19]. A 2D array consisting of a large number of simple repeating DNA tiles is filled in a hollow origami frame, which have faster growth kinetics when in an origami frame than when there is no frame. The design of DX molecules has been modified to obtain different DNA building blocks, such as paranemic crossover (PX) DNA, topoisomer (JX) DNA, and triple crossover complex. Through the strand displacement reaction, PX and JX can be converted into one another, that is, one end of a DNA strand rotates 180° relative to the other end. Seeman used this conformational change between PX and JX to confirm that a rotary nanomechanical device can be recycled [20].

A simple DX structure can be expanded into a high-order DNA motif, which can be assembled into a more complex 2D array, as shown in Figure 1e. Yan et al. designed a 4 × 4 DNA tile that can self-assemble into uniform-width nanoribbons, 2D nano−grids and ladder-like grids [10]. These grids can be subsequently used for the periodic arrangement of protein molecules and gold nanoparticles [26,27,28]. In addition, they also confirmed that sophisticated 2D and 3D tessellation patterns can be formed by using three- and four-arm DNA junction tiles with specifically designed arm lengths and inter-tile sticky-end interactions [29]. Mao et al. designed a series of n-pointed-stars and assembled them into 2D arrays with different topologies [23,24,30,31,32]. In addition, five- and six-pointed-stars have been co-crystallized to a 2D array similar to a quasi-crystal arrangement [33]. By controlling the flexibility and concentration of motifs, a DNA n-pointed-star motif can be assembled into a complex polyhedron framework structure (Figure 1d) [11,31], which can then be used as a nanocage to encapsulate nanoparticles in the frame to form clusters with a specific conformation [34,35,36,37]. Another complex motif formed by simple DNA tiles is tensegrity triangle. Mao et al. used three four-arm junctions to construct a tensegrity triangle with a double helix on each side [30] with sticky ends at each vertex, allowing it to self-assemble into one-dimensional (1D) or 2D ordered arrays by selectively using the vertices. Seeman et al. designed a tensegrity triangle composed of DX molecules, and used their assembled 1D and 2D arrays to complete the regular arrangement of gold nanoparticles (Figure 1e) [25]. Subsequently, Seeman et al. designed a tensegrity triangle with triple rotational symmetry in sequence [10]. The structure was assembled with sticky ends to obtain a single crystal with a rhombohedral lattice (Figure 1e). By adjusting the length of the sides of the tensegrity triangle, single crystals with different lattice parameters were obtained with cavities of different sizes, and the cavities could be used to accommodate guest particles. This work is considered to be a major breakthrough in the field of DNA nanotechnology. Since then, a lot of work has been carried out around the tensegrity triangle, such as controlling the crystal nucleation and growth process [38], improving the quality and stability of the single crystal [39,40], and completing dynamic changes [41].

The self-assembly product of DNA tiles can be used for the manipulation of nanoparticles. However, due to the limitation of DNA tile assembly products, nanoparticles can only be manipulated on a linear or planar template. The particle spacing and arrangement can be well controlled and, the polyhedral frame assembled by tiles can be used as a nanocage for nanoparticle encapsulation or as a restriction frame template to guide nanoparticle assembly. Thus, clusters of nanoparticles can be obtained, but it is difficult to expand them into a 3D space to form ordered crystals.

## 3. Self-Assembly Based on DNA Brick

**Figure 2:**
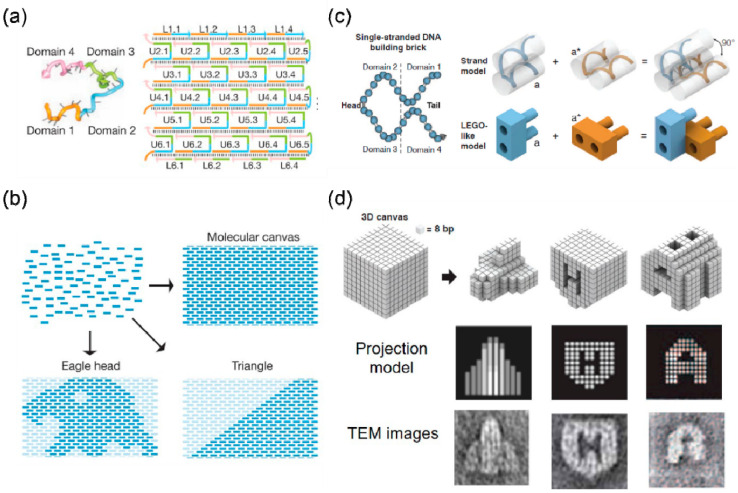
Single-stranded DNA tiles and single-stranded DNA bricks. (**a**) Rectangular 2D molecular canvas assembled by single-stranded DNA tiles. Reproduced from [42]. (**b**) Drawing the desired shape on the 2D molecular canvas. Reproduced from [42]. (**c**) LEGO model analogue of single-stranded DNA. Reproduced from [7]. (**d**) “Carving” the 3D canvas into any shape. Reproduced from [7].

The results of electron microscopy characterization indicate that the size and shape of a 2D array assembled by the DNA tile mentioned above cannot be controlled. Wei et al. proposed a “single-stranded DNA tile” (SST) that consists of a 42-base strand of DNA composed entirely of concatenated sticky ends. Therefore, each SST has the ability to bind with four local neighbors during self-assembly (Figure 2a) [42].

SST tiles can be assembled into a rectangular 2D array as a molecular canvas, where each SST DNA sequence is different and corresponds to a particular pixel in the molecular canvas (Figure 2a). The expected shape is drawn on the molecular canvas to find the target SST corresponding to all pixels in the shape, and then the target SST can be annealed in one pot to obtain the desired shape (Figure 2b). In this article, 107 unique and complex 2D shapes were synthesized in this way. DNA bricks are an extension of the concept of SST, extending the modular-assembly from 2D to 3D. Ke et al. first proposed the concept of DNA brick and used this method to construct more than 100 different 3D structures, as shown in Figure 2b,c [7]. Each brick is a single-stranded DNA with a distinct nucleic acid sequence. Utilizing the twist of the DNA helix, the complementary base pairing between adjacent bricks produces a dihedral angle close to 90 degrees (Figure 2c). Many bricks self-assemble into a 3D cubic molecular canvas onto which can be “carved” a variety of different 3D shapes (Figure 2d). Through one-pot annealing, the DNA strands corresponding to the bricks self-assemble to form the pre-designed 3D shape. DNA bricks provide a simple and modular method to assemble simple short DNA strands into complex 3D shapes. Later, Ke et al. constructed a 2D crystal with complex 3D geometric features (with the thickness dimension much smaller than the other two dimensions) and used it for the regular arrangement of gold nanoparticles [43]. The team later designed a second-generation brick with a longer bonding domain—because of which, it can self-assemble to form a larger and more complex 3D structure [12].

The largest advantage of DNA bricks is that the same batch of DNA bricks can be carved into any shape. In addition, since the DNA sequence of each brick is different, the composed 3D structure can be used as an addressable template to place the guest particles with a finer degree of control. Ke et al. also realized the single-layer and dispersed arrangement of gold nanoparticles on the 2D materials assembled by bricks [43]; however, the formation of multi-layer patterns of gold nanoparticles remains a challenge.

## 4. DNA Origami Assembly

**Figure 3:**
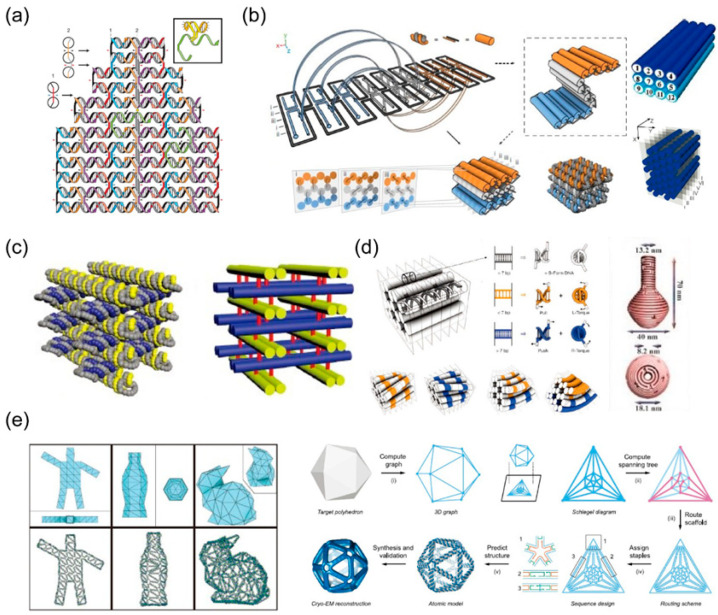
Development of DNA origami. (**a**) The origin of DNA origami: A long DNA strand is folded into a target structure through several short DNA strands. Reproduced with permission of [8]. Copyright Springer Nature, 2006. (**b**) Multilayer 3D DNA origami packed into a honeycomb structure. Reproduced with permission of [46]. Copyright Springer Nature, 2009. A square. Reproduced with permission of [47]. Copyright American Chemical Society, 2009. Hexagonal lattices. Reproduced with permission of [48]. Copyright American Chemical Society, 2012. (**c**) 3D DNA origami framework with layered crossovers. Reproduced with permission of [49]. Copyright WILEY−VCH Verlag GmbH & Co. KGaA, Weinheim, Germany, 2016. (**d**) Introducing twist and curve into DNA origami by deleting and adding bases. Reproduced with permission of [50,51]. Copyright American Association for the Advancement of Science, 2011. (**e**) The design of 3D wireframe DNA origami. Reproduced with permission of [53]. Copyright Springer Nature, 2015. Reproduced from [52].

In 2006, Rothemund proposed a strategy involving the folding of a long, single-stranded DNA (scaffold) into a desired single-layered, planar pattern (Figure 3a) [8]. This can be achieved by fixing the scaffold in place by hundreds of short oligonucleotides “staple strands”. Accordingly, other groups have successfully constructed arbitrary complex structures, for example a map of China and a depiction of a dolphin [44,45]. Then, Shih and his co-workers extended this method for constructing 3D structures (Figure 3b) [46]. Layer of helices were stacked to form a honeycomb lattice with increasing rigidity. In a similar way, multilayer DNA origami packed on a square and hexagonal lattice was constructed for improving the packing density [47,48]. Furthermore, Yan reported another novel design strategy to design a layered 3D framework of DNA structures by introducing layered crossover. In this design, the layered crossover permitted the scaffold or staple to go through different layers and controlled the relative directions of the DNA helices in the adjacent layers (Figure 3c) [49]. To create more complex structures, the twist and binding can be introduced by base pair insertions/deletions to adjust the number of base pairs between the crossover junctions, as shown in Figure 3d [50,51]. Another method to construct 3D DNA origami structures is to convert the target shapes into polygonal meshes and to apply a design algorithm to fully automate scaffold routing (Figure 3e) [52,53]. In addition, scaffold strand has been folded into six connected origami square sheets to create a 3D DNA cubic box with a dynamic “lid” [54].

However, the scale of DNA origami nanostructure is limited by the length of the scaffold. DNA origami as building blocks to form well-ordered structure can increase the scale of said structures. Additionally, the construction of crystal-like structures can be realized by hierarchical self-assembly. There are two strategies for hierarchically scaling up crystal DNA origami structures. The first strategy is to sequence specific base pairing between sticky ends that extend from origami monomers. Unique base sequences connect DNA origami monomers by recognizing each other through Watson Crick base pairing. The second strategy is the non-sequencing of specific base stacking by the blunt ends of the DNA helices. The origami monomers can be connected together by the blunt ends that occupy the edge of DNA origami. The interactions between DNA monomers can be more distinct by applying geometric coding to the sides of DNA origami. Both of these two methods (sticky end base pairing and blunt end base stacking) can be applied for building higher-order self-assembled structures.

### 4.1. Sticky End Base Pairing

**Figure 4:**
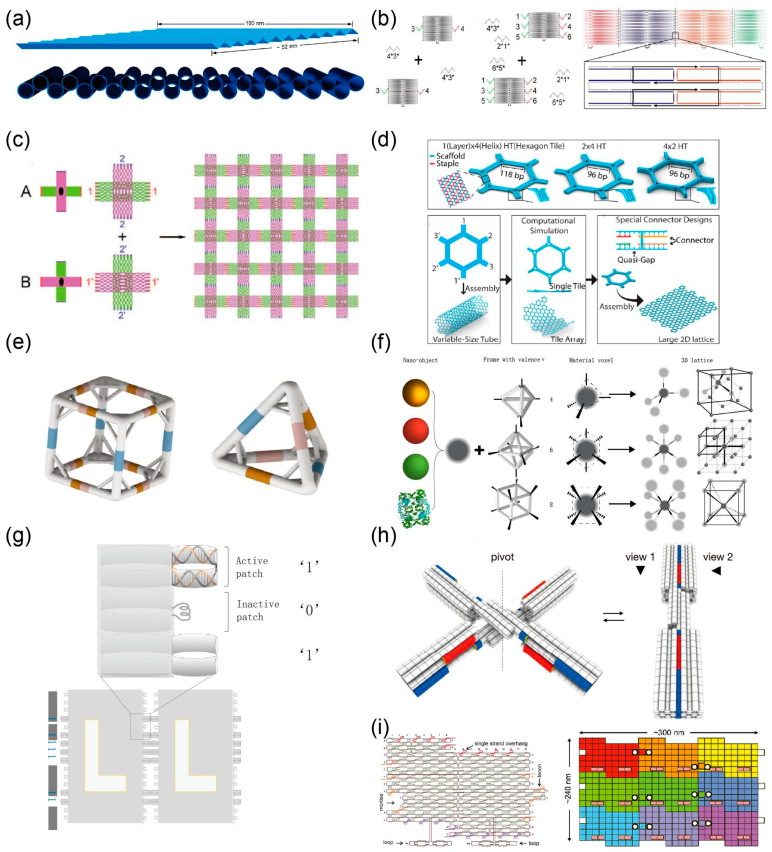
Sticky ends base pairing (**a**–**f**). (**a**) “Zigzag DNA origami” tiles were assembled into a one-dimensional (1D) lattice. Reproduced with permission of [55]. Copyright American Chemical Society, 2010. (**b**) Three different assembly methods of DNA origami monomers. Reproduced with permission of [56]. Copyright Springer Nature, 2011. (**c**) Assembly of a 2D DNA origami lattice using a cross-shaped origami tile. Reproduced with permission of [57]. Copyright WILEY−VCH Verlag GmbH & Co. KGaA, Weinheim, Germany, 2011. (**d**) Programming DNA origami honeycomb 2D lattices. Reproduced with permission of [58]. Copyright American Chemical Society, 2016. (**e**) A polyhedron self-assembled from DNA tripods. Reproduced from [59]. (**f**) Constructing different 3D DNA origami lattices using DNA-prescribed and valence-controlled material voxels. Reproduced with permission of [2]. Copyright Springer Nature, 2020. Blunt ends base stacking (**g**–**i**). (**g**) Recognition based on the binary sequences of blunt ends and the complementarity of the origami edge shapes of DNA nanostructures. Reproduced with permission of [60]. Copyright Springer Nature, 2011. (**h**) Self-assembly of 3D DNA components in a solution on the basis of shape complementarity. Reproduced from [61]. (**i**) Assembly of multiple DNA origami jigsaw pieces in three different ways. Reproduced with permission of [62]. Copyright American Chemical Society, 2011.

Two-dimensional crystalline DNA origami structures can be obtained by sticky end hybridization. Intermolecular contact can be established by programming Watson Crick complementary interactions. When constructing latticed DNA structures, many important factors need to be considered, such as the position and strength of the connectors and the global twist of the structure. One important factor in Rothemund’s original DNA origami design is that the interval of periodic crossovers between parallel helices are odd numbers of half-turns (for example, 16 bp for 1.5 turns) which results in a twist density of 10.67 bp per turn, while B-form DNA has a helical twist of 10.5 bp per turn. The increase in base pairs per turn in the design can cause a significant global twist. When hundreds or thousands of monomers are assembled, small distortions accumulate because of the global twist. Yan’s group constructed a family of rectangular-shaped DNA origami tiles containing 120°dihedral angles when viewed along the helical axes which is called “zigzag DNA origami” (Figure 4a) [55]. This design can avoid an inherent global twist in the primary planar, rectangular origami tiles. Then, origami tiles are assembled into different 1D lattice by designing several sticky ends. Jungmann et al. investigated three different methods for sequence-specific polymerization of origami monomers (Figure 4b) [56]. The first method is to extend a single strand of 10 bases at each short edge of the rectangular origami box. By designing a complementary linker strand of 20 bases, the monomers are connected to form a ribbon. The second method is similar to the first method, except that each monomer extends three different single sequences on each side, which are used to complement the extension sequences to connect monomers. The third method is to use a 32-nt-long “bridging strand” to connect two monomers. Twenty-four bases of ‘bridging strands’ are complementary to the scaffold of one monomer, and the remaining eight bases are complementary to the scaffold of the other monomer. The most effective method regarding yield and ribbon length is the third method which is the direct linkage of origami monomers by staple strands bridging the edges of the structures.

Liu et al. constructed a cross-shaped origami tile with two domains that DNA helices have in the orthogonal propagation direction (Figure 4c) [57]. They assembled large 2D lattices through sticky ends located at the end of the tiles. Small cross-shaped DNA motifs may have a certain curvature, so a corrugated design was required to create a planar 2D array, which can avoid the accumulation of curvature in any of the two dimensions during the crystal growth process. Ke and co-workers designed a set of hexagonal DNA-origami tiles, and constructed the self-assembly of micron-scale 2D honeycomb lattice and tubes via controlling their mechanical and geometric properties (including interconnecting strands) (Figure 4d) [58]. They found that a few factors play important roles in the results of DNA origami assembly. The bending or twisting of the tile itself helps to accumulate out-of-plane curvature, thereby forming a tube. In the case of minimum intrinsic curvature or twisting within the tile, the high stiffness of the tile is generally better able to resist the accumulation of out-of-plane curvature. They also investigated the effect of changing the design of the connection (such as the base pair length and the gap between connecting region) on a lattice formation. When additional base pair length reduces free energy, the lattice tends to form tubes. Therefore, it is generally considered that stronger sticky ends are advantageous for forming tubes. Introducing unpaired scaffold bases into the connector sequence may reduce the bonding strength of the connecting regions. In addition, unpaired scaffold bases can help mitigate the accumulation of curvature caused by twisting.

The method of sticky ends base pairing can also be extended to 3D crystalline DNA origami structures. Yin’s group reported a universal strategy that used stiff DNA origami “tripod” monomers with precisely controlled arm lengths and inter arm angles for the hierarchical self-assembly of polyhedrons (Figure 4e) [59]. A set of polyhedral structures was composed using tripod monomers with different angles, from tetrahedrons composed of four monomers to hexagonal prisms composed of 12 monomers. Tian et al. employed polyhedral frames to construct 3D DNA origami lattices via vertex-extended sticky ends hybridization (Figure 4f) [2]. They constructed different valence DNA frames, such as octahedral, cubic, and tetrahedral structures, and different nanomaterials were loaded inside through the hybridization of complementary strands. Material nano-objects and three-dimensional DNA frameworks with a prescribed valence were used to compose material voxels to form simple cubic, body-centered cubic, and diamond lattice structures.

### 4.2. Blunt Ends Base Stacking

Base stacking is the main force to stabilize a double-stranded DNA structure, but it is often overlooked when assembling higher-order DNA structures. Rothemund et al. showed that DNA can create varied bonds using the geometric arrangement of blunt-end stacking interactions (Figure 4g) [60]. In order to take advantage of the stacking force, a group of base stacking interactions can be programmed into binary or shape codes to achieve specific hybridization. In order to realize a binary sequence, the edge of a rectangular DNA origami is divided into many small patches. Patches with protruding blunt end doublets that allow stacking represent “1,” while non-protruding patches that contain a single scaffold loop that avoid stacking represent “0.” As for shape coding, the edge of the interface of DNA origami is divided into three patches of different depths; when the two sides match exactly, the DNA bases are stacked.

Gerling et al. recently used the rule of shape complementarity to specifically self-assemble discrete 3D DNA components in a solution (Figure 4h) [61]. They showed that the assembly of these origami units is stabilized by the base stacking bond against the electrostatic repulsion between component interfaces. By adjusting the experimental parameters, such as salt concentration and temperature, the interaction between the origami units can be switched on or off. The mechanism of the switch control is that the base stacking of complementary shapes is very sensitive to concentration of ions and temperature of solution. Both of these factors affect the attraction and repulsion between negatively charged DNA. By changing the Mg^2+^ concentration and temperature, the balance between base stacking force and electrostatic repulsion of DNA can be controlled and adjusted. Therefore, the open or closed state of DNA “origami” molecules can be dynamically changed, for example, from a compact DNA lattice structure to a grid-like lattice structure. Sugiyama et al. designed a group of DNA origami structures that can be expanded in both the horizontal and vertical directions using three connection methods, as shown in Figure 4i [62,63,64]. The four edges of DNA origami are attached with sticky ends, and the edges have π-stacking interactions in the horizontal (spiral axis) direction. At the same time, they also designed a jigsaw shape-complementary connection method through tenon and mortise structures. Using these different jigsaw DNA monomers, a cross-shaped, hollow square and 3 × 3 assembly structure was constructed.

## 5. Conclusions

Before the advent of DNA nanotechnology, it seemed impossible to construct such a complex structure through bottom-up self-assembly. However, in the field of DNA nanotechnology, researchers have successfully proved that DNA is not only the genetic material in cells, but also a powerful building material in the nano fields. The ability to hybridize complementary DNA strands provides programmability between DNA molecules. DNA nanotechnology uses this programmability of DNA molecules to design customized complex structures with high addressability. Based on the above discussion, the characteristics of the three methods (i.e., DNA tiles, DNA bricks, DNA origami) are listed in Table 1. These three DNA self-assembly methods all take advantage of the programmability of DNA molecules to achieve precision control over materials. Utilizing the advantages of each method has produced fruitful research results. For example, DNA nanotechnology has been widely used in the creation of dynamic nanostructures, such as DNA walkers [65], machines [66,67], and nanoscale assembly lines [68]. DNA structures based on tiles or origami have been introduced to cellular applications, including drug delivery [69] and bioimaging [70].

Despite these exciting achievements, DNA nanotechnology is still in its infancy, and there are many challenges and opportunities in this field. First, although some computer software systems can be used to design and verify the final structure, it remains difficult to obtain satisfactory results for some complex designs. An ideal software should be specifically designed for DNA nanotechnology. The trend is to construct larger and more complex DNA nanostructures; therefore, the software simplifies and speeds up the design process by providing easy-to-operate and top-to-down design options. Second, large-scale assembled DNA nanostructures are largely limited to a range of 100 nm to 10 μm, which limits their practical applications. Therefore, advanced design techniques are necessary to further improve the complexity, scale, and functionality of such structures. Third, the current experimental research typically focuses on small-scale structural designs, most of which are affordable. However, it becomes a problem when the structure reaches the sub-micron and micron scales. Recent work on DNA synthesis in biotechnology has revealed a potential method that can reduce the cost of DNA construction [71]. This strategy utilizes self-cleaving deoxyribozymes to automatically process DNA from ssDNA amplified by biotechnology, and has been confirmed on some origami structures. However, the versatility of this strategy needs to be further verified by different design techniques. Finally, the physical conditions including insufficient cationic ions and large amounts of DNA nuclease, greatly decline the stability of DNA nanostructures-although recent studies have shown that certain DNA nanostructures can withstand a period of time in an environment with a low ion concentration [72]. In the past few years, DNA nanotechnology has been promoted from simple systems to advanced applications, regardless of the existing challenges. DNA nanotechnology has the potential to bring huge changes to human manufacturing. When DNA is combined with inorganic materials, it can offer unprecedented precision and programmability to create metamaterials, superior plasmonic structures and nano-electronic circuits. DNA scaffolds serve as a “printing press” to transfer patterns to inorganic materials, so that the patterns are robust enough for applications. Moreover, when DNA is assembled with proteins, there arises very interesting opportunities in cell signaling and cell–cell interactions for biomedical utilizations. Constructing protein-mimicking DNA structures can provide a deeper understanding of protein function and can help to design more diverse functions exhibited by proteins. Moreover, DNA nanostructures offer incomparable opportunities for drug delivery, biophysics and biomedical applications. DNA or RNA devices may be able to function in vivo for sensing a set of signals on a biochemical pathway in the future. It is believed that researches evolving towards these applications will be conducted in the next few years that these applications will be used in both the clinic and field.

## Figures and Tables

**Table 1:** Characteristics of the three self-assembly methods.

Self-Assembly Method	DNA Tiles	DNA Bricks	DNA Origami
Starting Materials	Several Short Single-Stranded Oligonucleotides	A Single Short Oligonucleotide	Hundreds of Short Single-Stranded Oligonucleotides and A Long Oligonucleotide
Strategies of Scale-Up	Sticky Ends Base Pairing	Sticky Ends Base Pairing	Sticky Ends Base Pairing and Blunt Ends Base Stacking
Resulting Assembly	2D Array or 3D Polyhedral Frame	Arbitrary and Discrete 3D DNA Structures	1D, 2D or 3D Arbitrary Structures
Assembly Dimension	Up to 1 mm	Approximately 100 nm	Approximately 10 μm
